# Effect of *Nigella sativa* Supplementation on Oxidative Stress and Antioxidant Parameters: A Meta-Analysis of Randomized Controlled Trials

**DOI:** 10.1155/2020/2390706

**Published:** 2020-05-06

**Authors:** M. Ardiana, B. S. Pikir, A. Santoso, H. O. Hermawan, M. J. Al-Farabi

**Affiliations:** ^1^Medical Doctoral Program Student, Faculty of Medicine, Surabaya, Indonesia; ^2^Department of Cardiology and Vascular Medicine, Faculty of Medicine, University of Airlangga, Surabaya, Indonesia; ^3^Department of Cardiology, Faculty of Medicine, University of Indonesia, National Cardiovascular Centre, Harapan Kita Hospital, Jakarta, Indonesia; ^4^Postgraduate School of Biomedicine, Faculty of Medicine, Brawijaya University, Malang, Indonesia; ^5^Postgraduate School of Management, University College London, Gower St, Bloomsbury, London WC1E 6BT, UK

## Abstract

**Introduction:**

*Nigella sativa* is a commonly used traditional medicine which has been shown to have antioxidant properties. However, its supplementation in patients of clinical trials showed conflicting results. *Materials and Method*. Relevant articles were searched through PubMed/Medline, SCOPUS, and Google Scholar databases using “*Nigella sativa*” or “black seed” or “black caraway” or “thymoquinone” and “oxidative stress” or “antioxidant” and “clinical trial” keywords. Randomized, placebo-controlled human interventions using *Nigella sativa* were included in this study. The methodological quality of studies was assessed using Jadad's quality scales.

**Results:**

Five studies using 293 subjects met the inclusion criteria. The overall quality of all included trials was determined based on the low risk of bias and the high quality of reported information (Jadad score ≥ 3). Meta-analysis of 293 eligible subjects showed that treatment with *Nigella sativa* improved the superoxide dismutase (SOD) level (48.18; 95% CI 30.29 to 66.08; *p* < 0.01), but there was no significant effect on the malondialdehyde (MDA) level (−5.32; 95% CI −1.19 to 0.128; *p*=0.114) and total antioxidant capacity (TAC) level (0.219; 95% CI −0.136 to 0.573; *p* = 0.227).

**Conclusion:**

This meta-analysis suggests that *Nigella sativa* supplementation in humans may benefit as an antioxidant by increasing SOD levels but has no significant effect on the MDA level and TAC level.

## 1. Introduction

*Nigella sativa* (NS), which is commonly called black caraway, is a traditional medicine that has been used in Middle Eastern countries, Asia, Southern Europe, India, Pakistan, Syria, Saudi Arabia, Turkey, and the Southern Mediterranean countries. NS is a herbal plant from the family *Ranunculaceae* (buttercup), which contains many therapeutic benefits such as a bronchodilator, gastroprotective, hepatoprotective, antitumor, antidiabetic, antihypertensive, antioxidant, antifungal, immunomodulatory, anti-inflammatory, analgesic, antiviral, antipyretic, contraceptive, antimicrobial, anticonvulsant, antitussive, anticancer, antihyperlipidemic, and antibacterial effects [1–5]. The bioactive contents of NS are thymoquinone with derivatives such as dithymoquinone, thymohydroquinone, and thymol (30–48%), p-cymene (7%–15%), carvacrol (6%–12%), 4-terpineol (2%–7%), t-anethole (1%–4%), sesquiterpene longifolene (1%–8%), safranal, *α*-thujene, thymol, *α*-pinene, and *α*-terpinene which have been shown to have therapeutic effect for various diseases [6–9].

Oxidative stress is known to be the basis of the pathophysiology of several types of diseases such as coronary heart disease, chronic obstructive pulmonary disease, neurodegenerative diseases, cancer, and chronic kidney disease. Several studies have shown that NS has been proven to have antioxidant capabilities by reducing the production of reactive oxygen species (ROS) and malondialdehyde (MDA) [10, 11]. NS has also been shown to contain a high level of thymoquinone which can act as a free radical scavenger. NS is even able to increase the production and activity of antioxidant enzymes such as superoxide dismutase (SOD), catalase (CAT), glutathione peroxidase (GPx), and glutathione S transferase (GSH-ST) [12].

Clinical trials using NS supplementation showed conflicting results in the parameters of oxidative stress and antioxidant activity in the patients. Previous researches showed that supplementation of NS would significantly decrease the production of MDA, NO, and SOD in patients compared to controls [13–15]. However, other studies failed to find significant changes in the MDA, GPx, and total antioxidant capacity (TAC) [13, 15–17]. There is also no previous meta-analysis that summarized the antioxidant capabilities of NS supplementation. Therefore, this systematic review and meta-analysis is conducted to investigate the effects of NS supplementation on oxidative stress parameters such as MDA and the production of antioxidant enzymes represented by SOD and total antioxidant capacity (TAC) particularly in a randomized controlled trial (RCT). Thus, the finding may help the decision-making process of a physician as to the usage of NS supplementation to improve antioxidant status.

## 2. Method

The data in this meta-analysis are reported according to the guidelines of Preferred Reporting Items for Systematic Reviews and Meta-Analyses (PRISMA) [18].

### 2.1. Search Strategy

Relevant articles were searched through PubMed/Medline, SCOPUS, and Google Scholar databases using keywords including “Nigella sativa” or “black seed” or “black caraway” or “thymoquinone” and “oxidative stress” or “antioxidant” and “clinical trial” or “clinical” or “patient.” No restriction was conducted based on language or publication date. To avoid missing any study, the reference lists of all eligible articles, related reviews, and meta-analyses were also reviewed. The references cited by all the selected original research articles and reviews were searched for additional articles that might have been missed. Any document reporting a measure of NS supplementation in clinical trials based on primary data was considered. Studies were excluded if they did not contain primary data, were on younger patients of nonspecified age, or used unreferenced sources. Duplicate data, commentaries, letters, correspondence, and editorials are excluded. Unpublished records such as conference papers, theses, and patents were not included in this meta-analysis.

### 2.2. Eligibility Criteria

Studies were selected if they met these criteria: (1) full-text publication written in English; (2) randomized controlled trials (either parallel or crossover); (3) conducted on ≥18 year-old subjects; (4) evaluate the effects of NS on oxidative parameters and/or antioxidant enzymes; (5) reported parameters before and after the intervention in both placebo and treatment groups.

### 2.3. Exclusion Criteria

The exclusion criteria of this meta-analysis were as follows: (1) being nonclinical trials studies; (2) conducted on children, pregnant women, or animals; (3) not being placebo-controlled trials; (4) lack of sufficient data for the outcomes of interest in NS or control group; (5) examining the effect of NS supplementation along with other interventions.

### 2.4. Data Extraction

Two independent reviewers extracted data. The following data were extracted from the included studies by using standardized protocol: first author's name; year of publication; study location; study duration; age and gender of participants; study design; health status of study population; number of participants in each groups; type and dose of NS supplements; and mean ± standard deviation (SD) and *p* value of oxidative stress and antioxidant enzyme parameters. MDA was measured in nmol/L, SOD was measured in Unit/mL, and TAC was measured in mM.

### 2.5. Quality Assessment of Studies

Each paper's quality was independently assessed by two experts and disagreements were resolved through consensus. The Jadad score system was used to assess the quality of included studies. This scale consisted of five questions; a score of 0 or 1 is given to each question on (i) randomization, (ii) appropriate method for randomization, (iii) double-blinding, (iv) appropriate method for double-blinding, and (v) description of dropouts and withdrawals. Each publication will have an overall score of 0–5, with higher scores representing better methodological quality. Publications with a score of >3 were considered of high quality, whereas those with lower scores were defined as low-quality studies.

### 2.6. Statistical Analysis

NS supplementation and control group will be pooled and evaluated for the mean differences and standard deviations (SDs) of the following outcomes: (i) MDA, (ii) SOD, and (iii) TAC. The fixed-effect model is used to estimate the overall effect size for homogeneous data. The random-effect model is used to estimate the overall effect size for heterogeneous data. Heterogeneity was examined using Cochrane's *Q* test (significance point at *p* < 0.05). The risk of bias in reporting the cumulative incidence was independently calculated by the authors. The publication bias of each study was assessed through funnel Egger's test [19]. All statistical analyses were done using Stata software version 12 (StataCorp, College Station, Texas, USA). *p* < 0.05 was considered as statistically significant.

### 2.7. Ethical Approval

This meta-analysis did not require ethical approval since all of the data retrieved for the studies were already available in the public domain.

## 3. Results

### 3.1. Study Selection

**Figure 1:**
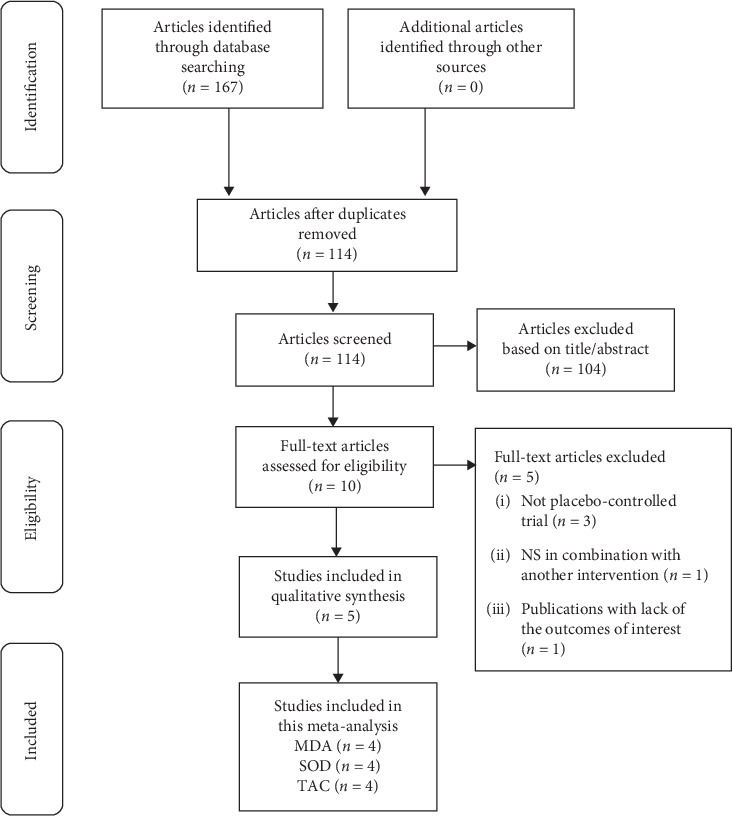
Flow diagram of the number of studies included in the meta-analysis.

Overall, five eligible RCTs were included in this meta-analysis. Among them, two articles reported the effect of *Nigella sativa* on MDA, TAC, and SOD [14, 15]; one article reported the effect of *Nigella sativa* on MDA and SOD [13]; one article reported the effect of *Nigella sativa* on MDA and TAC [16]; and one article reported the effect of *Nigella sativa* on SOD and TAC [17]. The flow diagram for the study selection is shown in Figure 1.

At the initial search, we found 167 relevant records. After removing duplicates (*n* = 53), 114 records remained and were reviewed based on title and abstract. Therefore, 10 eligible studies are selected for a careful full-text assessment. Studies that were not placebo-controlled trial (*n* = 3), combined NS with other interventions (*n* = 1), and did not evaluate any of the MDA, TAC, and SOD (*n* = 1) were excluded.

### 3.2. Study Characteristics

The characteristics of the included studies are indicated in Table 1. These studies had enrolled a total of 293 subjects (women and men), 152 subjects in the NS group and 141 in the control group, with an age range of 18–60 years. They were published between 2015 and 2019. From these studies, 4 were conducted in Iran [13–16] and the other was in Saudi Arabia [17]. All trials involved in this research were considered to have high quality (Jadad score ≥3) [13–17].

The studies used NS doses of 500 mg/day [13, 14, 16], 2 g/day [17], or 3 g/day [15], with an intervention duration of 6 weeks [16], 8 weeks [13–15], and 48 weeks [17]. Three trials were carried out on women [13–15] and the others on both genders [16, 17]. Patients with type 2 diabetes [14, 17], rheumatoid arthritis [13], obesity [15], and ulcerative colitis [16] participated in these studies. The effect of NS supplementation was compared with placebo in four studies while the study of Namazi et al. compared the effect of a low-calorie diet with or without NS supplementation in the intervention and placebo-controlled groups [15]. All included studies were parallel-design clinical trials. Significant reduction in MDA was reported in one study after intake of NS, as compared with placebo [16], while changes in the MDA level were not significant in the other RCTs [13–15, 17]. One study found a significant increase in TAC in the NS group compared to controls [17]. However, other studies showed no significant differences [13–16]. Conflicting results were also found regarding the SOD level. Two studies showed an increase of SOD in the NS group compared to controls [14, 17] while two other studies showed a reduction of SOD in the NS group compared to controls [13, 15]; however, the effect was only significant in one study [15].

### 3.3. Effect of NS Supplementation on MDA

**Figure 2:**
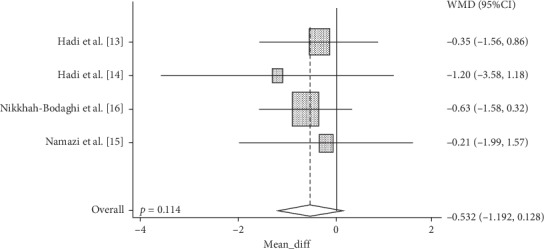
Forest plot displaying weighted mean difference and 95% confidence intervals for the impact of NS supplementation on MDA.

Cochrane's *Q* test showed that the MDA data was homogeneous (*p*=0.906). Hence, the fixed-effect model method was used to create a forest plot. Analysis from four studies showed that NS supplementation did not significantly reduce MDA (Weighted Mean Differences (WMD): −0.532, 95% CI: −1.192 to 0.128, *p*=0.114)) compared to controls (Figure 2).

### 3.4. Effect of NS Supplementation on SOD

**Figure 3:**
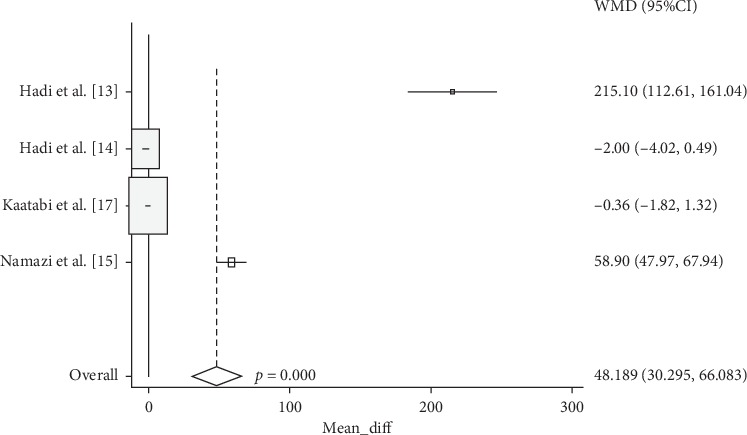
Forest plot displaying weighted mean difference and 95% confidence intervals for the impact of NS supplementation on SOD.

Cochrane's *Q* test showed that the data is heterogeneous (*p*=0.01). Hence, the random-effect model method was used to create a forest plot. Analysis from four studies showed that NS supplementation significantly increases SOD (Weighted Mean Differences (WMD): 48,189, 95% CI: 30.295 to 66.083, *p*=0.01)) as compared to controls (Figure 3).

### 3.5. Effect of NS Supplementation on TAC

**Figure 4:**
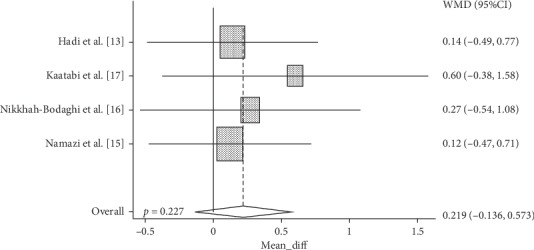
Forest plot displaying weighted mean difference and 95% confidence intervals for the impact of NS supplementation on TAC.

Cochrane's *Q* test showed that the data is homogeneous (*p*=0.857). Hence, the fixed-effect model method was used to create a forest plot. Analysis from four studies showed that NS supplementation did not significantly increase TAC (Weighted Mean Differences (WMD): 0.219, 95% CI: −0.136 to 0.573, *p*=0.227)) as compared to controls (Figure 4).

## 4. Discussion

Oxidative stress is caused by the imbalance of free radicals and the body's antioxidant defenses [10]. Failure to defend against free radicals will cause lipid peroxidation which is marked by the production of MDA. MDA levels have been known to increase various diseases associated with oxidative stress [11]. In this review, we found no significant changes in the MDA level for subjects that received NS supplements compared to placebo; only a single study showed a significant reduction in MDA [16], whereas the other studies showed no significant differences [13–15]. The possible explanation of this result may be the differences in treatment duration. A significant reduction of MDA was observed in the NS supplementation for six-week duration [16] while in all other researches NS was supplemented for eight weeks [13–15]. Additionally, this might be caused by the great data range and standard deviation in all four studies involved.

Interestingly, several nonplacebo clinical trials on NS supplementation showed a significant effect of MDA reduction after NS supplementation for 12 weeks [20, 21]. When compared with the preclinical studies, NS treatments also consistently decrease MDA levels in the rats treated with carbon tetrachloride [22], diabetic rats [23, 24], lipopolysaccharide-treated rats [25], mice treated with reactive peroxyl [26], hyperlipidemic rabbits [7], and other various studies. Hence, the significant changes of MDA levels in the NS supplementation may be dependent on various variables such as the duration of NS supplementation, different cellular conditions, and different disease types and severity. However, further research is required to validate these hypotheses. We did not adjust these factors because of the limited number of available studies.

An antioxidant is defined as a substance that can inhibit oxidative processes and inhibit chemical reactions that transfer electrons or hydrogen to oxidizing agents. There are two kinds of antioxidant systems which are enzymatic such as superoxide dismutase (SOD), catalase (CAT), and glutathione peroxidase (GSH-Px) and nonenzymatic antioxidant systems such as uric acid, vitamin C, vitamin E, glutathione, bilirubin, *α*-lipoic acid, and carotenoids [27]. Total antioxidant capacity (TAC) is defined as the cumulative effect of all antioxidants in the blood and body fluids [28]. In this meta-analysis, SOD levels were found to be increased significantly. This study showed that SOD levels increased significantly with NS supplementation. When compared to preclinical studies, NS treatments also consistently significantly increased the SOD level in the rats treated with potassium bromate [29], cadmium [30], diazinon [31], lead [32], and formaldehyde [33] which induce oxidative stress. Previously, it is also suggested that the major reduction of free radicals in the treatment with NS was mainly due to the increase of SOD [12]. SOD can convert free radical superoxide anions to hydrogen peroxide and oxygen, thus reducing cellular damage and disease progression, which was worsened by oxidative stress [34]. Interestingly, in this meta-analysis, the effect of NS supplementation on TAC was not significant, suggesting that although the effect of NS is beneficial for increasing enzymatic antioxidants such as SOD, the overall effect of NS on the TAC might not be beneficial.

NS contains a high amount of thymoquinone which has been proven to enhance the enzyme function in lipid metabolism and protect cells against lipid peroxidation [1]. Thymoquinone from NS has been shown to be able to increase the SOD activity [35]. This suggested that thymoquinone may be responsible for the beneficial effect of NS supplementation on increased SOD levels in this research. Other than thymoquinone, flavonoids, phenolic content, and vitamins such as ascorbic acid in NS may also contribute as antioxidants. The NS was also proven to improve the nonenzymatic antioxidant system through direct scavenging of carbon-centered radicals and hydroxyl radicals [35, 36].

To the best of our knowledge, this is the first meta-analysis that evaluates the effect of NS supplementation on oxidative stress and antioxidant parameters. However, some limitations existed in this meta-analysis. Several factors that cannot be standardized between studies may greatly affect the meta-analysis results. These factors include nonstandardized NS formulation since there is a lack of regulation for nutraceutical products, in addition to the difference in dose, disease type and severity, and supplementation duration between research studies. Therefore, further clinical trials with similar intervention period, similar type of disease and severity, more subjects, and standardized NS preparation are needed. In conclusion, this meta-analysis shows a significant effect of NS supplementation on the increase of SOD, but not on MDA and TAC.

## Figures and Tables

**Table 1:** Characteristics of the included studies.

Author	Year	Study design	Target population	NS form	Dose/day	Duration	Gender	Age	Sample size	Outcome	Jadad score
Hadi et al. [14]	2018	Randomized, double-blind, placebo-controlled trial	DM type 2	Oil capsule	500 mg	8 weeks	Female	30–60	43	MDA, SOD, TNF-*α*, IL-1*β*, CAT, NO	3

Hadi et al. [13]	2016	Randomized, double-blind, placebo-controlled trial	Rheumatoid arthritis	Oil capsule	500 mg	8 weeks	Female	20–50	39	MDA, TAC, SOD, TNF-*α*, IL-10, CAT, NO	4

Kaatabi et al. [17]	2015	Randomized, participant blind, placebo-controlled trial	DM type 2	Powder capsule	2 gr	48 weeks	Both	18–60	114	TAC, SOD, FBG, HbA1c, C-peptide, insulin resistance, beta-cell activity, CAT, glutathione, TBARS	3

Nikkhah-Bodaghi et al. [16]	2019	Randomized, double-blind, placebo-controlled trial	Ulcerative colitis	Powder capsule	500 mg	6 weeks	Both	>18	48	MDA, TAC, TNF-*α*, hs-CRP, NFkB	4

Namazi et al. [15]	2015	Randomized, double-blind, placebo-controlled trial	Obese	Oil capsule	3 gr	8 weeks	Female	25–50	49	MDA, TAC, SOD, GPx	4

## Data Availability

The data used to support the findings of this study are available from the corresponding author upon request.
